# Flattening Filter-Free Beams in Intensity-Modulated Radiotherapy and Volumetric Modulated Arc Therapy for Sinonasal Cancer

**DOI:** 10.1371/journal.pone.0146604

**Published:** 2016-01-06

**Authors:** Jia-Yang Lu, Jing Zheng, Wu-Zhe Zhang, Bao-Tian Huang

**Affiliations:** 1 Department of Radiation Oncology, Cancer Hospital of Shantou University Medical College, Shantou, Guangdong, China; 2 Department of Laboratory, Shantou Central Hospital, Affiliated Shantou Hospital of Sun Yat-sen University, Shantou, Guangdong, China; Taipei Medical University, TAIWAN

## Abstract

**Purpose:**

To evaluate the dosimetric impacts of flattening filter-free (FFF) beams in intensity-modulated radiotherapy (IMRT) and volumetric modulated arc therapy (VMAT) for sinonasal cancer.

**Methods:**

For fourteen cases, IMRT and VMAT planning was performed using 6-MV photon beams with both conventional flattened and FFF modes. The four types of plans were compared in terms of target dose homogeneity and conformity, organ-at-risk (OAR) sparing, number of monitor units (MUs) per fraction, treatment time and pure beam-on time.

**Results:**

FFF beams led to comparable target dose homogeneity, conformity, increased number of MUs and lower doses to the spinal cord, brainstem and normal tissue, compared with flattened beams in both IMRT and VMAT. FFF beams in IMRT resulted in improvements by up to 5.4% for sparing of the contralateral optic structures, with shortened treatment time by 9.5%. However, FFF beams provided comparable overall OAR sparing and treatment time in VMAT. With FFF mode, VMAT yielded inferior homogeneity and superior conformity compared with IMRT, with comparable overall OAR sparing and significantly shorter treatment time.

**Conclusions:**

Using FFF beams in IMRT and VMAT is feasible for the treatment of sinonasal cancer. Our results suggest that the delivery mode of FFF beams may play an encouraging role with better sparing of contralateral optic OARs and treatment efficiency in IMRT, but yield comparable results in VMAT.

## Introduction

Sinonasal cancers (SNCs) are uncommon, accounting for only 3–5% of all head and neck malignancies [1–3]. They are typically diagnosed at locally advanced stages, where surgical operation and postoperative radiation therapy represent the standard of care [4,5]. Over the last decade, intensity-modulated radiotherapy (IMRT) and volumetric modulated arc therapy (VMAT) have become prevalent treatment techniques for SNCs [6–8], owing to their dosimetric advantages along with the clinical preservation of nearby optic structures [9–11] while maintaining disease control and survival. However, treatment planning for SNC is challenging due to the proximity and/or involvement of multiple critical organs at risk (OARs) including the optic nerves, optic chiasm, lenses, brain, parotid glands and brainstem. Making compromises is sometimes necessary in order to avoid overdosing the optic structures [12] or ensure target dose coverage. How to design radiotherapy plans for SNC remains an interesting investigative topic.

Conventional radiation beams from medical linear accelerators are flattened in order to generate a homogeneous dose distribution at a certain depth for an open treatment field, by inserting a flattening filter into the head of the linear accelerators. In recent years, there has been a growing interest in the removal of the flattening filter, which results in a flattening filter-free (FFF) beam. The FFF beams are characterized by high dose rate, cone-like fluence profile, softened beam quality [13], increased superficial dose, reduced out-of-field dose [14,15] and high dose calculation accuracy (at least as high as for flattened beams) [16]. Modern radiotherapy techniques, such as IMRT and VMAT, are able to generate intensity modulated beams using multi-leaf collimator (MLC) motion series in combination with inverse planning. Since the fluence profile can be taken into consideration during optimization, the conventional flattened beams become unnecessary in this situation. The clinical application of FFF beams has been investigated in many studies for the cases of breast cancer [17], lung cancer [18] and other tumor sites [19–23]. These studies concluded in general that the FFF beams resulted in similar plan qualities and reduction of treatment time. However, none of these studies has been focused on dosimetric roles of FFF beams in the SNC cases. As the FFF beams can deliver lower out-of-field dose, there might be some potential dosimetric benefits with respect to the sparing of lenses or other OARs. Therefore, we compared the FFF beams with conventional beams in the IMRT and VMAT for SNC in this study, aiming to identify the dosimetric effects of this delivery mode and selecting the reasonable radiotherapy technique for the treatment of SNC.

## Methods

### Ethics statement

The protocol was approved by the Ethical Commission of the Cancer Hospital of Shantou University Medical College. Because this was not a treatment-based study, our institutional review board waived the need for written informed consent from the participants. The patient information was anonymized and de-identified to protect patient confidentiality.

### Patient characteristics

Computed tomography (CT) scan datasets of 14 patients diagnosed as melanoma (Patients 1–3), esthesioneuroblastoma (Patients 4 and 5), squamous cell carcinoma (Patients 6–9), adenoid cystic carcinoma (Patient 10), sarcoma (Patient 11) and NK/T cell lymphoma (Patients 12–14) of the nasal cavity, maxillary sinus and ethmoid sinus were selected. The patients included 8 males and 6 females, with a median age of 62 years (range, 32–66 years). In accordance with the American Joint Committee on Cancer (AJCC) Seventh Edition staging system, the patients were at stage T2-T4, N0 and M0. All the patients received surgical operations followed by postoperative radiotherapy except for the 3 NK/T cell lymphoma patients who received radiotherapy alone.

### CT simulation and the delineation of target and OARs

All the patients were immobilized in supine position in a tailor-made head-neck-shoulder thermoplastic cast. CT scans with a 3-mm slice thickness were performed using a 16-slice CT scanner (Philips Brilliance CT Big Bore Oncology Configuration, Cleveland, OH, USA). The CT images were then transferred to the Eclipse^TM^ version 10.0 treatment planning system (Varian Medical System, Inc., Palo Alto, CA) for target and OAR delineation and treatment planning.

All target volumes were delineated by our radiation oncologists. Gross tumor volume (GTV) was defined as the visible extent of tumor identified utilizing contrasted CT, MR and positron emission tomography (PET) for definitively treated patients. The clinical target volume (CTV) comprises the primary tumor bed and the zones at risk of harboring microscopic extension. The planning target volume (PTV) was derived from the clinical target volume plus a uniform 5-mm margin, and was then cropped 3 mm away from the surface of the body to avoid the parts extending outside the body and the build-up effect. The median volume of the PTV was 185 cubic centimeters (cc) with a range of 102–259 cc.

The OARs included the lenses, optic nerves, optic chiasm, eyes, spinal cord, brainstem, temporal lobes, cochleae, pituitary, oral cavity and parotids. The “PTV_in_skin” was generated from the portion of PTV within a ring structure generated by a 7-mm inner margin of the body [20]. Surrounding normal tissue was defined as the body volume excluding the PTV.

### Linear accelerator calibration

A TrueBeam® (Varian Medical System, Inc., Palo Alto, CA) linear accelerator was used to deliver 6-MV FFF beams and conventional flattened beams. The output of both beams were calibrated such that 1 MU gave 0.01-Gy dose to water at central axis at a depth of maximum dose for a field size of 10 × 10 cm^2^ and for a source-to-surface distance (SSD) of 100 cm.

### Radiotherapy treatment planning

The IMRT plans using non-coplanar 6-MV FFF beams (FFF-IMRT) and conventional flattened beams (C-IMRT) from TrueBeam® were generated in Eclipse^TM^. The beam arrangement was set according to the study by Jeong *et al* [4] with minor modifications (Field 1/Field 2, gantry 260°/100° with collimator 330°/30° and couch 0°; Field 3/Field 4, gantry 330°/30° with collimator angles optimized to minimize the exposure to the lenses, with fixed jaw and with couch 0°; Field 5, gantry 0° with collimator 0° and couch 0°; Field 6/Field 7, gantry 330°/30° with collimator 0° and couch 90°). The VMAT plans with 6-MV FFF beams (FFF-VMAT) or conventional flattened beams (C-VMAT) were generated using two coplanar arcs of 360° with collimators rotated to 30° and 330°, respectively to minimize the tongue and groove effect. Maximum dose rates of 600 and 1400 monitor units (MUs)/minute were selected for the conventional flattened and FFF beams, respectively. Prescription doses were set to 60 Gy (2 Gy/fraction) administered in 30 fractions for both IMRT and VMAT. Optimizations were performed with the Dose Volume Optimizer (DVO, version 10.0.28) and Progressive Resolution Optimizer (PRO, version 10.0.28) algorithms for IMRT and VMAT, respectively. The Anisotropic Analytical Algorithm (AAA, version 10.0.28) was applied for final dose calculations, with a grid size of 2.5 mm. Dose-limiting ring structures were generated to form the dose gradients surrounding the PTV. Each treatment plan was normalized such that 95% of the PTV received the prescribed dose of 60 Gy.

The same optimization objectives were adopted for the FFF-IMRT, C-IMRT, FFF-VMAT and C-VMAT plans. The IMRT plans were further optimized utilizing Eclipse^TM^’s “base dose plan” function to improve the plan qualities. The “base dose plan” function enabled the system to optimize a plan (as a second plan) while taking another plan (as a base dose plan) into account, aiming to achieve an optimal plan sum by making up for inadequacies (hot/cold spots) in the base dose plan. Our approach utilizing the “base dose plan” function is described briefly as follows: with optimization objectives being unmodified, the treatment plan duplicated from the original plan with half of total fractions was further optimized based on the original plan with half of total fractions, and then the number of fractions of the treatment plan was restored from a half to the total. The details of this approach applied in head-and-neck cancer were introduced in our previous study [24]. The VMAT plans were further optimized once or twice to improve the plan qualities. Treatment planning goals are listed in Table 1. D_x%_ represents the dose which is reached or exceeded in x% of the volume and V_xGy_ represents the % volume receiving a dose of x Gy. D_2%_ and D_98%_ represent the near-maximum and near-minimum doses, respectively according to the International Commission on Radiation Units and Measurements (ICRU) report 83 [25]. D_mean_ represents the mean dose. The optimization objectives were adjusted to ensure that the D_2%_ of PTV was below the 110% of the prescription dose. The sparing of lenses, optic chiasm and optic nerves was set to the highest priority with the aim of preserving at least unilateral vision, followed by the PTV coverage objectives. The sparing of brainstem and spinal cord was set to the third priority, and the dose limitations of the remaining OARs and ring structures were set to the last priority.

**Table 1:** Treatment planning goals for sinonasal cancer.

Structure	Planning constraint(s)
**PTV**	D_95%_ = 60 Gy
	D_2%_ < 66 Gy (110% of the prescription dose)
**Lens**	D_2%_ < 10 Gy
**Optic nerve**	D_2%_ < 54 Gy
**Optic chiasm**	D_2%_ < 54 Gy
**Eye**	D_2%_ < 50 Gy
**Spinal cord**	D_2%_ < 40 Gy
**Brainstem**	D_2%_ < 50 Gy
**Temporal lobe**	D_2%_ < 60 Gy
**Cochlea**	D_5%_ < 55 Gy, D_mean_ < 45 Gy
**Pituitary**	D_2%_ < 60 Gy
**Oral cavity**	D_mean_ < 30 Gy
**Parotid**	D_50%_ < 30 Gy, D_mean_ < 26 Gy
**Normal tissue**	As low as possible

PTV = planning target volume; D_x%_ = dose that is reached or exceeded in x% of the volume; D_mean_ = mean dose.

All the plans were conducted by one medical physicist to avoid individual variation. The numbers of MUs per fraction were compared. The treatment time which included the gantry and couch rotation time but excluded the patient setup time was recorded. Additionally, the pure beam-on time of the linear accelerator was also recorded. The treatment efficiency was defined as the treatment task completed by the linear accelerator per unit of treatment time. The treatment efficiency is inversely proportional to the treatment time [26].

### Plan evaluation

Dose-volume statistics, isodose distributions and cumulative dose-volume histograms (DVHs) were computed to compare the plans. D_2%_ and D_98%_ were selected for the appraisals of hot and cold spots, respectively. The target dose homogeneity was quantified using the homogeneity index (HI) recommended by the ICRU report 83 [25]. The target dose conformity was measured using the conformity index (CI) proposed by Paddick [27].

### Statistical analysis

To determine the statistical significance of the differences among the techniques, two-tailed paired Wilcoxon signed-rank tests were performed with a *P*-value of < 0.05 considered to be significant, using SPSS version 19 software (SPSS, Inc., Chicago, IL, USA).

## Results

### Target coverage, homogeneity and conformity

**Fig 1:**
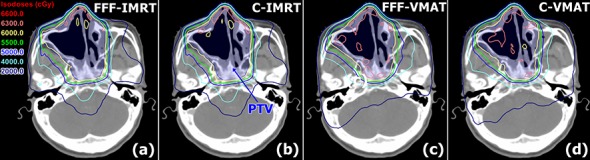
Dose distributions of the flattening filter-free intensity-modulated radiotherapy (FFF-IMRT), conventional IMRT (C-IMRT), flattening filter-free volumetric modulated arc therapy (FFF-VMAT) and conventional VMAT (C-VMAT) plans for Patient 4.

All the PTVs received sufficient dose coverage. For each plan, the D_95%_ of PTV was normalized to 60 Gy and the D_2%_ of the PTV was lower than 66 Gy. The data for the PTV (Table 2) demonstrate that the D_2%_ values, D_98%_ values, HIs and CIs were comparable between the FFF beams and conventional flattened beams both for IMRT and VMAT (*P* > 0.05), and the D_98%_ of PTV_in_skin was increased by 0.9% with FFF beams in IMRT. When compared to FFF-IMRT, FFF-VMAT yielded 1% higher D_2%_ and 0.7% lower D_98%_ for the PTV, and produced inferior HI by 29.7% and superior CI by 2.7%. In the isodose distribution, fewer hot spots of ≥ 105% (63 Gy) of the prescribed dose for the PTV were observed for IMRT (Fig 1).

**Table 2:** Dosimetric parameters for the flattening filter-free intensity-modulated radiotherapy (FFF-IMRT), conventional IMRT (C-IMRT), flattening filter-free volumetric modulated arc therapy (FFF-VMAT) plans and conventional VMAT (C-VMAT).

		FFF-IMRT	C-IMRT	FFF-VMAT	C-VMAT	*P*-value		
						FFF-IMRT *vs* C-IMRT	FFF-VMAT *vs* C-VMAT	FFF-IMRT *vs* FFF-VMAT
**PTV**	**D**_**2%**_ **(Gy)**	62.98 ± 0.69	62.90 ± 0.76	63.63 ± 0.98	63.53 ± 0.76	0.330	0.490	0.001
	**D**_**98%**_ **(Gy)**	59.40 ± 0.25	59.34 ± 0.25	58.96 ± 0.29	58.96 ± 0.29	0.064	0.878	0.001
	**D**_**50%**_ **(Gy)**	61.26 ± 0.27	61.26 ± 0.31	61.96 ± 0.68	61.88 ± 0.48	0.889	0.124	0.001
	**HI**	0.058 ± 0.014	0.058 ± 0.016	0.075 ± 0.019	0.074 ± 0.016	0.875	0.470	0.001
	**CI**	0.869 ± 0.019	0.865 ± 0.016	0.892 ± 0.020	0.896 ± 0.016	0.245	0.074	0.003
**PTV_in_skin**	**D**_**98%**_ **(Gy)**	56.48 ± 1.19	55.97 ± 1.39	56.64 ± 0.70	56.57 ±0.76	0.016	0.258	0.433
**CL lens**	**D**_**2%**_ **(Gy)**	6.50 ± 1.31	6.84 ± 1.21	7.95 ± 0.95	7.96 ± 0.85	0.002	0.778	0.001
**IL lens**	**D**_**2%**_ **(Gy)**	8.98 ± 2.15	9.10 ± 2.15	8.78 ± 1.59	8.92 ± 1.50	0.363	0.030	0.451
**CL optic nerve**	**D**_**2%**_ **(Gy)**	43.85 ± 9.69	44.48 ± 9.63	49.50 ± 4.86	49.41 ± 5.08	0.103	0.683	0.005
**IL optic nerve**	**D**_**2%**_ **(Gy)**	52.09 ± 4.06	52.16 ± 3.55	54.20 ± 3.26	53.70 ± 3.62	0.683	0.011	0.001
**Optic chiasm**	**D**_**2%**_ **(Gy)**	45.40 ± 6.95	45.82 ± 6.57	44.06 ± 10.42	44.81 ± 9.65	0.198	0.026	0.510
**CL eye**	**D**_**2%**_ **(Gy)**	33.62 ± 12.86	34.93 ± 13.43	36.43 ± 8.50	34.62 ± 8.81	0.008	0.002	0.074
**IL eye**	**D**_**2%**_ **(Gy)**	46.75 ± 6.30	46.89 ± 5.54	46.48 ± 5.15	45.55 ± 5.48	0.433	0.022	0.510
**Spinal cord**	**D**_**2%**_ **(Gy)**	15.08 ± 5.36	15.56 ± 5.57	9.13 ± 6.11	10.29 ± 6.91	0.001	0.001	0.001
**Brainstem**	**D**_**2%**_ **(Gy)**	37.96 ± 4.88	38.23 ± 4.68	28.10 ± 8.89	29.58 ± 8.21	0.045	0.013	0.002
**CL temporal lobe**	**D**_**2%**_ **(Gy)**	37.84 ± 10.57	37.78 ± 9.30	38.64 ± 7.95	38.94 ± 8.08	0.158	0.433	0.594
**IL temporal lobe**	**D**_**2%**_ **(Gy)**	49.76 ± 6.61	50.43 ± 6.17	45.61 ± 7.12	46.07 ± 6.82	0.510	0.397	0.003
**CL cochlea**	**D**_**5%**_ **(Gy)**	24.15 ± 13.68	24.53 ± 14.13	28.05 ± 8.55	28.52 ± 8.35	0.221	0.510	0.177
	**D**_**mean**_ **(Gy)**	18.93 ± 10.81	19.05 ± 10.73	25.22 ± 6.99	25.53 ± 6.60	0.245	0.594	0.019
**IL cochlea**	**D**_**5%**_ **(Gy)**	33.32 ± 7.84	33.89 ± 8.33	31.34 ±7.61	33.57 ± 7.97	0.638	0.048	0.140
	**D**_**mean**_ **(Gy)**	27.78 ± 6.63	27.75 ± 6.93	27.35 ± 5.42	29.57 ± 6.11	0.875	0.030	0.594
**Pituitary**	**D**_**2%**_ **(Gy)**	44.06 ± 9.41	44.41 ± 9.02	40.16 ± 12.54	41.45 ± 11.74	0.084	0.074	0.026
**Oral cavity**	**D**_**50%**_ **(Gy)**	16.83 ± 6.77	16.67 ± 6.67	8.11 ± 8.75	8.41 ± 8.38	0.245	0.026	0.002
	**D**_**mean**_ **(Gy)**	22.27 ± 5.76	22.29 ± 5.78	16.73 ± 6.38	16.98 ± 6.21	0.594	0.022	0.002
**CL parotid**	**D**_**50%**_ **(Gy)**	5.03 ± 6.53	5.05 ± 6.39	7.14 ± 7.67	7.06 ± 6.97	0.109	0.315	0.003
	**D**_**mean**_ **(Gy)**	6.02 ± 5.13	6.07 ± 5.05	8.94 ± 5.91	8.81 ± 5.38	0.060	0.975	0.002
**IL parotid**	**D**_**50%**_ **(Gy)**	7.98 ± 6.12	8.04 ± 6.10	8.69 ± 7.02	8.97 ± 7.02	0.233	0.069	0.875
	**D**_**mean**_ **(Gy)**	9.03 ± 5.72	9.07 ± 5.68	10.90 ± 6.22	11.03 ± 6.22	0.300	0.551	0.004
**Normal tissue**	**V**_**5Gy**_ **(%)**	39.2 ± 10.9	40.7 ± 11.4	31.8 ± 10.1	32.4 ± 10.4	0.001	0.001	0.001
	**V**_**10Gy**_ **(%)**	22.3 ± 6.9	22.9 ± 7.1	23.5 ± 7.1	24.8 ± 7.6	0.001	0.001	0.054
	**V**_**20Gy**_ **(%)**	10.7 ± 3.2	10.9 ± 3.3	11.9 ± 3.3	12.2 ± 3.4	0.002	0.009	0.002
	**V**_**30Gy**_ **(%)**	5.6 ± 1.8	5.8 ± 1.8	6.4 ± 1.7	6.3 ± 1.7	0.006	0.085	0.001

PTV = planning target volume; CL = contalateral; IL = ipsilateral; D_x%_ = dose which is reached or exceeded in x% of the volume; V_xGy_ = volume receiving at least x-Gy dose; HI = homogeneity index; CI = conformity index; D_mean_ = mean dose.

### OAR sparing

The doses delivered to all the OARs, except the ipsilateral lens and optic nerve that were in close proximity to or a part of the PTV, were limited to the tolerance levels. As shown in Table 2, FFF-IMRT allowed additional D_2%_ reductions of 5.4%, 3.2%, 3.0% and 0.8% with regards to the contralateral lens, contralateral eye, spinal cord and brainstem, respectively compared with C-IMRT. FFF-IMRT also gave smaller V_5Gy_, V_10Gy_, V_20Gy_ and V_30Gy_ of normal tissue by 1.4%, 0.6%, 0.2% and 0.2%, respectively. When compared with C-VMAT, FFF-VMAT provided lower D_2%_ to the ipsilateral lens, optic chiasm, spinal cord and brainstem, by 1.7%, 2.2%, 9.8% and 5.5%, respectively, but delivered higher D_2%_ to the ipsilateral optic nerve, contralateral eye and ipsilateral eye by 1.0%, 5.8% and 2.2%, respectively. With respect to the normal tissue, minor improvements with FFF beams were observed in terms of V_5Gy_, V_10Gy_ and V_20Gy_ by 0.7%, 1.3% and 0.4%, respectively, along with similar V_30Gy_.

**Fig 2:**
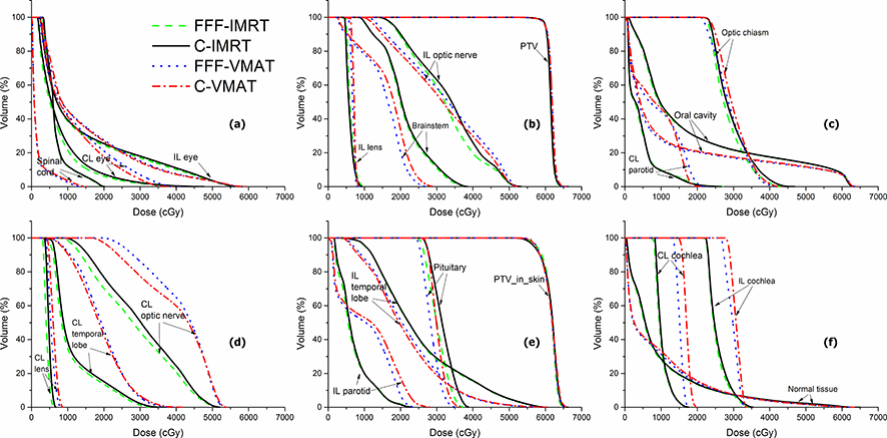
Dose-volume histograms (DVHs) of the flattening filter-free intensity-modulated radiotherapy (FFF-IMRT), conventional IMRT (C-IMRT), flattening filter-free volumetric modulated arc therapy (FFF-VMAT) and conventional VMAT (C-VMAT) plans for Patient 4.

As to the comparison of FFF-IMRT and FFF-VMAT, FFF-IMRT tended to deposit lower doses to most of the optic structures including the contralateral lens and bilateral optic nerves by 3.9%-18.4%, and displayed better sparing of the contralateral cochlea and bilateral parotids. However, FFF-VMAT exhibited significant dose reduction of the spinal cord, brainstem, ipsilateral temporal lobe, pituitary and oral cavity by 8.3%-45.0%. Concerning the normal tissue, smaller V_5Gy_ was identified for FFF-VMAT while smaller V_20Gy_ and V_30Gy_ were observed for FFF-IMRT (*P* < 0.05). These results are also illustrated in Fig 2 for Patient 4.

### MUs and delivery time

From the data presented in Table 3, increased number of MUs was observed for the use of FFF beams compared with conventional flattened beams, on average by 34.9% for IMRT and by 4.5% for VMAT. For IMRT, the FFF beams resulted in a decrease of beam-on time by an average of 42.2%, but the shorter beam-on time only translated into a reduction in the total treatment time by an average of 9.5%. For VMAT, no significant differences were found in terms of beam-on time and treatment time. Moreover, FFF-VMAT showed significant reductions of the MUs (by 66.3%) and treatment time (by 60.7%) compared to FFF-IMRT, although the pure beam-on time of FFF-IMRT was 55.0% less than that of FFF-VMAT.

**Table 3:** Delivery parameters for the conventional intensity-modulated radiotherapy (C-IMRT), flattening filter-free IMRT (FFF-IMRT), conventional volumetric modulated arc therapy (C-VMAT) and flattening filter-free VMAT (FFF-VMAT) plans.

	FFF-IMRT	C-IMRT	FFF-VMAT	C-VMAT	*P*-value		
					FFF-IMRT *vs* C-IMRT	FFF-VMAT *vs* C-VMAT	FFF-IMRT *vs* FFF-VMAT
**Monitor units**	1294 ± 172	964 ± 154	427 ± 23	409 ± 19	0.001	0.001	0.001
**Treatment time (minute)**	6.4 ± 0.2	7.0 ± 0.3	2.5 ± 0.0	2.5 ± 0.0	0.001	0.428	0.001
**Beam-on time (minute)**	0.9 ± 0.1	1.6 ± 0.3	2.1 ± 0.0	2.1 ± 0.0	0.001	0.066	0.001

## Discussion

As earlier published studies [6,8] have demonstrated, no significant dosimetric differences were observed between non-coplanar VMAT and coplanar VMAT for SNC, thus we only investigated the coplanar VMAT in this study for its advantage of less positioning uncertainty. In general, our data have implied that the FFF beams may provide encouraging results for the IMRT of SNC and comparable overall results for VMAT. For IMRT, the FFF beams reduced the doses to the contralateral lens, contralateral eye, spinal cord, brainstem and normal tissue, and improved the treatment efficiency. For VMAT, the FFF beams decreased the doses to spinal cord and several other OARs, but also increased the doses to the ipsilateral optic nerve and bilateral eyes, and maintained equivalent treatment efficiency. When the comparison of FFF-IMRT and FFF-VMAT is considered, FFF-IMRT obtained superior homogeneity and better sparing of contralateral optic structures and parotids, whereas FFF-VMAT had superior conformity and better sparing of several other structures.

Our finding that target dose coverage, conformity and homogeneity were comparable between FFF beams and conventional flattened beams in both IMRT and VMAT is similar to numerous other studies [17–19,22,23]. In modern radiotherapy techniques, the non-uniform dose distribution from a single open field of FFF beams can be compensated for by the increasing number of MUs which deposit dose at certain distances from beam’s central axis where FFF fields deliver less dose per MU than flattened fields owing to the conical profile [17,18,20,23]. In addition, as the minimum dose of tumor predominately correlates with the tumor control probability (TCP) [28], the higher near-minimum dose to PTV_in_skin with FFF beams may have a positive impact on the TCP for the cases with superficial PTV. The relatively higher superficial dose is caused by the softened beam quality of FFF beams with elimination of the hardening effect of flattening filter. The percentage depth dose (PDD) distribution of 6-MV energy FFF beams was previously found to be close to that of conventional flattened 4-MV energy beams by Vassiliev *et al*’s study [13]. With regard to VMAT, though the anterior gantry angles could deliver a higher dose to PTV_in_skin, but the lower dose delivered to PTV_in_skin by posterior gantry angles counteracted this effect, resulting in similar doses between FFF-VMAT and C-VMAT. Moreover, our results showed that FFF-IMRT provided better dose uniformity than FFF-VMAT did, which is different from the results of other researches [4,6,7]. The explanation is that we used the special optimization approach mentioned above to improve our IMRT plan qualities [24]. This approach utilized the dose of the initial IMRT plan as a base dose for further optimization to compensate for the systematic optimization-convergence error [29], and as a result, the hot and cold spots were substantially reduced and the homogeneous dose distribution was achieved.

Our finding that the involvement of the contralateral lens and contralateral eye was significantly reduced by FFF-IMRT confirmed our conjecture and is in accordance with the characteristic of lower out-of-field dose. To our knowledge, none of the previous studies [17,19,21–23] has reported the sparing effect of FFF beams for IMRT, which may bring some potential clinical benefits to patients. The sparing of the optic pathway is crucial for the quality of life of the patients with long-term survival. Though Duprez *et al* [9] have concluded that the IMRT technique could minimize the ocular toxicity compared with conventional radiotherapy techniques, there were still 10 cases of late Grade 3 tearing and 1 case of late Grade 3 visual impairment in their group of 86 patients available for late toxicity evaluation. Similar studies were also presented in the review by Chi *et al* [10]. Furthermore, Ainsbury *et al* [30] suggested that radiation cataractogenesis may in fact be more accurately described by a linear, no-threshold model. Therefore, further reductions of doses to the optic structures are essential to obtain an optimal clinical outcome. On the other hand, FFF-VMAT showed inferior sparing of optic structures compared with FFF-IMRT and this may be attributed to the beam arrangement and fixed jaw technique aiming at minimizing the exposure to the lenses and other optic structures.

For both IMRT and VMAT, the FFF beams could reduce the doses deposited in the spinal cord and brainstem, which was expected to reduce the risks of radiation-induced myelitis and brainstem necrosis [31]. It could be beneficial to patients with locally residual or recurrent diseases, especially with a requirement of re-irradiation [32].

Our finding that the FFF beams reduced the V_5Gy_, V_10Gy_, V_20Gy_, V_30Gy_ to normal tissue by up to 1.4% is in favor of the research result presented by Nicolini *et al* [19], which found that FFF-VMAT reduced the V_10Gy_ of healthy tissue by approximately 0.8% compared with C-VMAT. This is because the FFF beams could reduce collimator scatter and head leakage and consequently reduced the out-of-field dose [15,33]. Since the secondary cancer risk is closely associated with the exposure of normal tissue and total body [34], the FFF beams’ efficacy of delivering lower dose to normal tissue and less head leakage may have a potential benefit of reducing the risk of secondary cancer, especially for young patients. However, a mitigating factor to this is the increased number of MUs of FFF plans, which would increase the tissue scatter from the treatment region.

Our result that the FFF beams obtained 9.5% reduction of treatment time and 42.2% reduction of beam-on time for IMRT is similar to Spruijt *et al*’s research [17]. They reported the 10% reduction of total treatment time and 31% reduction of beam-on time. Although the effect of the shortened treatment time is limited, FFF-IMRT would be more patient friendly and entail less likelihood of intrafraction shifts of tumor position. However, it is noteworthy that a few seconds of treatment time saved by the FFF beams can be thwarted because of a difference in patient setup time. When considering the VMAT technique, the treatment time required only 2.5 minutes in both FFF-VMAT and C-VMAT. The explanations of equal treatment/beam-on time for FFF-VMAT and C-VMAT were that the actual dose rates in both were around 200 MUs/minute, which were much lower than the maximum dose rates of 1400 and 600 MUs/minute selected, and the restricting factor of the treatment time was the gantry rotation, which already maintained a maximum speed of 6°/s during the dose delivery process.

To the best of our knowledge, our study is the first to report the impacts of FFF beams on the case of SNC. However, this is only a dosimetric study and a further study may be required to explore the clinical outcomes among these different techniques.

## Conclusion

For SNC treatment, the FFF beams yielded comparable target dose conformity, homogeneity, reduced normal-tissue doses and increased number of MUs compared with flattened beams in both IMRT and VMAT. The FFF beams demonstrated some improvements in contralateral optic structures and other structures as well as delivery efficiency in IMRT, whereas they provided comparable overall OAR sparing and delivery efficiency in VMAT. Our results suggest that using FFF beams in IMRT and VMAT is feasible for the treatment of SNC, and the delivery mode of FFF beams may play an encouraging role in IMRT, but yield comparable results in VMAT.
